# Bone health in women with hidradenitis suppurativa: addressing osteoporosis risks and management options

**DOI:** 10.1097/JW9.0000000000000262

**Published:** 2026-06-26

**Authors:** Gabriela E. Beraja, Ana Maria Kausel, Jenny E. Murase, Lindsey Bordone, Andrew Phillip Sawaya, Juan Pablo de Rivero Vaccari, Hadar Lev-Tov

**Affiliations:** a Dr. Phillip Frost Department of Dermatology and Cutaneous Surgery, University of Miami Miller School of Medicine, Miami, Florida; b Anzara Health, Miami, Florida; c Department of Dermatology, University of California, San Francisco, California; d Department of Dermatology, Palo Alto Foundation Medical Group, Mountain View, California; e Department of Dermatology, Columbia University Irving Medical Center, New York City, New York; f The Miami Project to Cure Paralysis, University of Miami Miller School of Medicine, Miami, Florida

**Keywords:** Comorbidities, hidradenitis suppurativa, osteoporosis, inflammation, menopause, screening

## Abstract

**Background:**

Hidradenitis suppurativa (HS) is a chronic inflammatory skin disorder characterized by painful nodules and abscesses in intertriginous areas, predominantly affecting women. Persistent systemic inflammation and hormonal changes may contribute to reduced bone health and an increased risk of osteoporosis in this population.

**Objective:**

To highlight osteoporosis as an underrecognized comorbidity in women with HS and discuss contributing inflammatory, hormonal, and lifestyle-related risk factors that may predispose patients to bone compromise.

**Methods:**

A narrative review of the literature was conducted examining the relationship between HS, chronic inflammation, menopause-related estrogen decline, and osteoporosis risk. Additional contributory factors commonly associated with HS, including tobacco use, vitamin D deficiency, obesity, physical inactivity, and repeated corticosteroid exposure, were evaluated.

**Results:**

Systemic inflammation, hormonal decline, corticosteroid exposure, vitamin D deficiency, and reduced activity may contribute to increased osteoporosis risk in women with HS.

**Limitations:**

Current evidence evaluating osteoporosis risk specifically in patients with HS remains limited, and additional prospective studies are needed to better characterize prevalence, mechanisms, and screening recommendations.

**Conclusion:**

Bone health should be considered in women with HS, particularly those with severe or longstanding disease.

**What is known about this subject in regard to women and their families?**

- Women, particularly those with hidradenitis suppurativa (HS), are at an increased risk of developing osteoporosis, especially during and after menopause because of the natural decline in estrogen.
- Women with long-standing HS may begin to lose bone density well before the standard screening age of 65, largely because of the ongoing inflammatory burden of the disease.
- Many families are unaware that HS is a systemic condition with impacts that extend beyond the skin to include metabolic complications such as weakened bones.
- Delays in osteoporosis screening are common, resulting in missed chances to detect and manage bone loss early in women at elevated risk.

**What is new from this article as messages for women and their families?**

- Osteoporosis should be recognized as a critical comorbidity in women with HS, not just an age-related condition.
- Screening should begin earlier than general population guidelines for women with severe or long-standing HS.
- Hormone replacement therapy, commonly used to address menopausal symptoms and preserve bone health, may worsen HS in some women, highlighting the need for alternative osteoporosis therapies.

Women are at an increased risk of osteoporosis primarily due to a significant decline in estrogen levels during the transition to menopause.^1^ Estrogen promotes osteoblast activity, inhibits osteoclasts, enhances calcium absorption in the intestines, and regulates bone remodeling.^2^ These processes involve various cytokines and growth factors that are crucial for bone metabolism.^2^ Starting at age 40 years, women begin to lose bone density at a rate of 0.3 to 0.5% per year, which accelerates to 3 to 5% per year during menopause, typically lasting for approximately 5 to 7 years.^3^

For women with chronic inflammatory conditions, persistent systemic inflammation further compounds their vulnerability to bone loss. HS is one such condition, characterized by elevated levels of proinflammatory cytokines such as tumor necrosis factor-α, interleukin (IL)-1β, and IL-17, which can impair osteoblast differentiation and activity.^4–8^ HS frequently coexists with other metabolic and inflammatory disorders, including diabetes, obesity, inflammatory bowel disease, and psoriasis, each of which independently contributes to increased osteoporosis risk.^9–11^

Lifestyle factors commonly observed in HS that further jeopardize bone health include smoking,^12^ vitamin D deficiency,^13^ and obesity.^14^ Smoking is particularly detrimental, as it impairs intestinal calcium absorption and accelerates age-related bone loss.^15^ Obese women typically have higher circulating levels of estrogen, which can offer some protection against osteoporosis; however, an elevated body mass index (BMI) is often associated with reduced physical activity, leading to insufficient mechanical loading on bones and an increased risk of osteoporosis.^16^

The treatments for acute HS, including the use of systemic glucocorticoids and intralesional steroid injections, may contribute to bone loss through cumulative corticosteroid exposure. Although systemic absorption from injections is generally low, systemic effects have been reported with chronic or high-dose use. A case series of patients receiving long-term intralesional triamcinolone acetonide for alopecia areata found reduced bone mineral density (BMD), particularly with cumulative doses exceeding 500 mg and in those with additional osteoporosis risk factors.^17^ Given that many patients with HS receive monthly intralesional steroid injections of 5 to 40 mg across multiple sites over extended periods, this cumulative exposure warrants consideration as a potential risk factor for bone loss. For example, at a concentration of 20 mg/mL,^18^ injecting 5 tunnels with 1 mL each delivers approximately 100 mg of triamcinolone acetonide per session, reaching a cumulative 500 mg after only 5 monthly treatments. This illustrates how patients with multiple active sites and ongoing disease activity can readily exceed doses associated with measurable systemic effects in other populations.

Navarro et al.^7^ were the first to examine bone health in individuals with HS in Spain, reporting significantly lower total hip BMD (0.934 ± 0.02 vs 0.988 ± 0.02 g/cm^2^; *P* = .047) and trabecular bone scores (TBS) (1.36 ± 0.01 vs 1.40 ± 0.01; *P* = .02) compared with age- and sex-matched controls. Dual-energy X-ray absorptiometry (DEXA) is the standard technique used to measure BMD, and TBS, derived from lumbar spine DEXA images, provides an indirect assessment of trabecular microarchitecture and complements BMD in assessing fracture risk.^19^ Similarly, Miller et al. conducted a cross-sectional study in Denmark involving 32 individuals with HS, revealing lower muscular and bone mass percentages in this population.^20^ Since HS can persist into later years, with late-onset affecting 0.2% and persistent HS affecting 0.8% of individuals over age 65 years, individuals with longstanding or severe disease may face compounded risks for bone health.^21^ As a result, earlier screening should be considered for patients with HS who have prolonged disease duration (≥10 years), high inflammatory burden, or additional risk factors such as postmenopausal status, low BMI, smoking, vitamin D deficiency, or chronic corticosteroid use, particularly before the standard age threshold for screening (ie, women ≥65 and postmenopausal women <65 with risk factors).^22^ Baseline laboratory evaluation should include complete blood count, complete metabolic panel, phosphate, serum 25-hyroxyvitamin D, and parathyroid hormone (PTH) levels, with additional testing (eg, thyroid stimulating hormone and 24-hour urine to test for calcium, creatinine, and sodium levels) as indicated to assess for secondary causes of bone loss.^23^

Other inflammatory skin conditions, such as psoriasis and atopic dermatitis, are associated with an increased risk of osteoporosis, driven primarily by chronic inflammation and corticosteroid use.^24^ A large study in the United States found psoriasis patients had nearly 3 times the risk of osteoporosis and higher fracture rates^25^ and a United Kingdom cohort study linked severe psoriasis to increased fracture risk.^26^ Atopic dermatitis patients also face an almost 5-fold higher incidence of osteoporosis.^27^ Although steroid treatment regimens differ, the shared inflammatory pathways among these conditions underscore the importance of evaluating bone health in HS.

Some women elect to improve bone health through hormone replacement therapy (HRT).^28^ However, the impact of HRT for HS remains unclear, as evidence on hormonal influences in the postmenopausal woman is limited and conflicting.^29^ A survey study by Fernandez et al. found that participants reported worsening (39.5%) or no change (44.2%) in HS symptoms after menopause,^30^ whereas Kromann et al. reported that nearly half of the women in their study (29 out of 61) experienced symptom alleviation during menopause.^31^ Because the course of HS during menopause is not well defined, it is difficult to predict how HRT might affect disease activity, and further research is needed.

In the absence of data on HRT safety or efficacy in HS, clinicians should focus on evidence-based strategies to preserve bone health. Agents like bisphosphonates (eg, alendronate 70 mg orally weekly), monoclonal antibodies (eg, denosumab 60 mg subcutaneously every 6 months, romosozumab 210 mg subcutaneously monthly), or PTH/PTH-related protein analogues (eg, teriparatide 20 µg subcutaneously daily) may be better management options.^32^ Additionally, lifestyle modifications such as regular weight-bearing exercises and smoking cessation, along with calcium (1,000–1,200 mg per day) and vitamin D supplementation (400–1,000 IU) to maintain serum 25-hydroxy vitamin D3 levels between 30 and 50 nmol/L, are crucial (Table 1).^2,31^ Resistance training combined with high-impact or weight-bearing exercises effectively preserves BMD at the femoral neck and lumbar spine.^34^ Clinicians should work with physical therapists to develop personalized exercise plans, with forthcoming data from ongoing trials to inform future recommendations.^35^

**Table 1:** Examples of pharmacologic and adjunctive therapies for postmenopausal osteoporosis^32,33^

Drug class	Medication name	Mechanism of action	Route	Doses
Bisphosphonates^a^	Alendronate	Inhibition of osteoclasts (inhibits bone resorption)	Oral	Prevention: 35 mg weekly or 5mg daily Treatment: 70 mg weekly or 10 mg daily
RANK ligand inhibitor	Denosumab	Human monoclonal antibody against receptor activator of nuclear factor-κB ligand (RANKL); inhibits osteoclast activity	SC	60 mg every 6 months
Sclerostin inhibitor	Romosozumab	Human monoclonal antibody against sclerostin; increases bone formation, reduces bone resorption	SC	210 mg monthly
PTH analogue	Teriparatide	Recombinant human parathyroid hormone (PTH) that increases osteoblastic activity; increases bone formation	SC	20 mcg daily for up to 2 years
Vitamin D		Promotes intestinal absorption and reduces renal excretion rate of calcium; increases mineralization of bones	Oral	400–1,000 IU daily (if levels are 30–50 nmol/L)
Calcium		Building block for synthesis of bone	Oral	1,000–2,000 mg daily

SC = subcutaneous.

a Preferred first-line agent

In conclusion, hormonal changes related to menopause, lifestyle and comorbid factors, and the use of steroid therapies for acute management all significantly affect women with HS, elevating their risk of bone deterioration. Dermatologists play an important role in identifying at-risk patients with HS and initiating early discussions about bone health. When appropriate, they should order baseline labs (complete blood count, complete metabolic panel, phosphate, serum 25-hyroxyvitamin D, and PTH) and DEXA/TBS imaging (Fig. 1) and/or coordinate referral to primary care or endocrinology for further evaluation. Individuals with HS should undergo osteoporosis screening at the onset of menopause or earlier (if ≥10 years of HS duration), high inflammatory burden, or additional risk factors such postmenopausal status, low BMI, smoking, vitamin D deficiency, or chronic corticosteroid use, particularly before the standard age threshold for screening (ie, women ≥65 and postmenopausal women <65 with risk factors). Treatment should be initiated promptly upon detection of osteopenia or osteoporosis. Given the complex interplay between hormonal changes, therapy for HS, and inflammation in HS, osteoporosis should be recognized as a critical comorbidity warranting further investigation. By prioritizing research and assessing bone health in patients with HS, we can improve overall patient care and develop comprehensive treatment strategies that address both inflammatory and metabolic concerns.

**Fig. 1.**
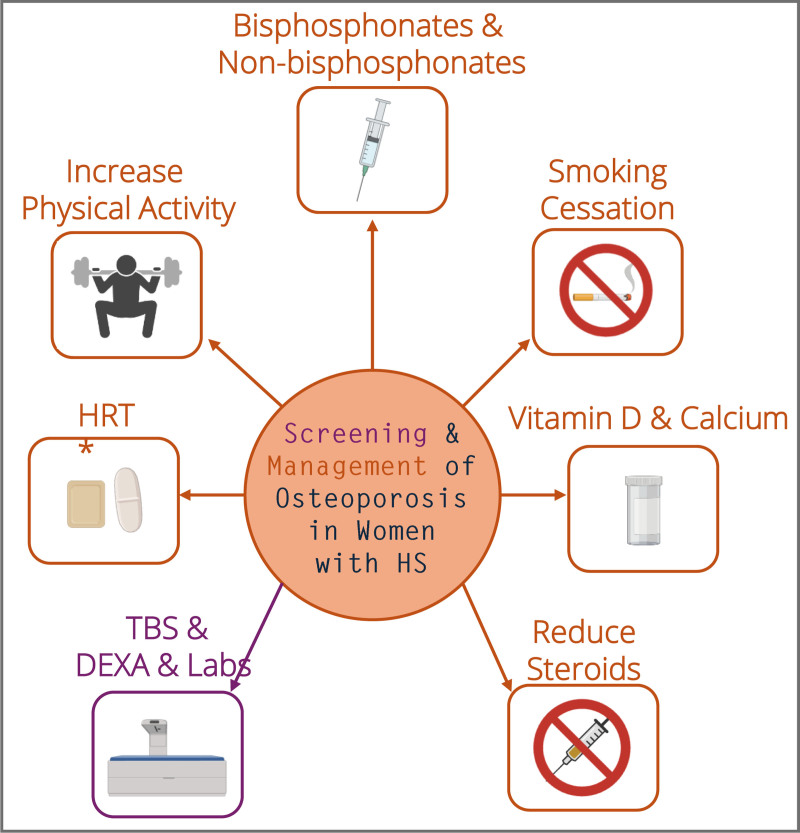
Screening and management of osteoporosis in women with hidradenitis suppurativa (HS). This figure outlines key strategies for osteoporosis prevention and treatment in women with HS, including lifestyle modifications (increased physical activity, smoking cessation, vitamin D/calcium supplementation, and steroid reduction), pharmacologic interventions (bisphosphonates and nonbisphosphonates), bone density screening using trabecular bone scan (TBS) and dual-energy X-ray absorptiometry (DEXA) scans, and laboratory evaluation. *Hormone replacement therapy (HRT) may be considered with caution. Symbols adapted from BioRender.com.

## Conflicts of interest

The authors made the following disclosures: J.E.M reports a relationship with Regeneron Pharmaceuticals Inc that includes board membership, consulting or advisory, and speaking and lecture fees; Sanofi Genzyme that includes board membership, speaking, and lecture fees; Galderma Laboratories that includes speaking and lecture fees; UCB Pharma that includes board membership, speaking, and lecture fees; Arcutis Biotherapeutics, Inc. that includes board membership; Bristol Myers Squibb Co that includes board membership; AbbVie Inc. that includes consulting or advisory; and UptoDate Inc. that includes consulting or advisory services. J.E.M. was the prior co-editor-in-chief of the *International Journal of Women’s Dermatology*. H.L.-T. reports a relationship with Learnskin.com that includes board membership and equity or stocks; Insmed Incorporated that includes consulting or advisory services; Next Science USA that includes consulting or advisory services; Novartis that includes consulting or advisory; UCB Pharma SA that includes consulting or advisory; Essity that includes nonfinancial support; Next Science USA that includes nonfinancial support; Sigavris that includes nonfinancial support; and Vomaris that includes nonfinancial support. J.P.R.V. reports a relationship with InflamaCORE, LLC that includes board membership. The remaining authors declare no conflicts of interest.

## Funding

None.

## Study approval

N/A

## Author contributions

GEB: Conceptualization, writing—original draft, writing—review and editing. AMK: Conceptualization, writing—review and editing. JEM: Conceptualization, writing—review and editing. LB: Writing—review and editing. APS: Writing—review and editing. JPRV: Writing—review and editing. HL-T: Supervision, writing—review and editing, project administration.
